# Regulation of Anti-*Plasmodium* Immunity by a *LITAF*-like Transcription Factor in the Malaria Vector *Anopheles gambiae*

**DOI:** 10.1371/journal.ppat.1002965

**Published:** 2012-10-18

**Authors:** Ryan C. Smith, Abraham G. Eappen, Andrea J. Radtke, Marcelo Jacobs-Lorena

**Affiliations:** Department of Molecular Microbiology and Immunology, Malaria Research Institute, Johns Hopkins Bloomberg School of Public Health, Baltimore, Maryland, United States of America; University of Minnesota, United States of America

## Abstract

The mosquito is the obligate vector for malaria transmission. To complete its development within the mosquito, the malaria parasite *Plasmodium* must overcome the protective action of the mosquito innate immune system. Here we report on the involvement of the *Anopheles gambiae* orthologue of a conserved component of the vertebrate immune system, LPS-induced TNFα transcription factor (*LITAF*), and its role in mosquito anti-*Plasmodium* immunity. *An. gambiae LITAF*-like 3 (*LL3*) expression is up-regulated in response to midgut invasion by both rodent and human malaria parasites. Silencing of *LL3* expression greatly increases parasite survival, indicating that *LL3* is part of an anti-*Plasmodium* defense mechanism. Electrophoretic mobility shift assays identified specific LL3 DNA-binding motifs within the promoter of *SRPN6*, a gene that also mediates mosquito defense against *Plasmodium*. Further experiments indicated that these motifs play a direct role in LL3 regulation of *SRPN6* expression. We conclude that *LL3* is a transcription factor capable of modulating *SRPN6* expression as part of the mosquito anti-*Plasmodium* immune response.

## Author Summary

The mosquito innate immune system serves as the primary defense response against invading pathogens, including that of the malaria parasite *Plasmodium*. The mosquito immune response is remarkably efficient in eliminating the parasite as indicated by the low prevalence of *Plasmodium* oocysts in wild caught mosquitoes. In an effort to understand the mechanisms of immune response, we report the first evidence of a LPS-induced TNF-α factor (*LITAF*)-like gene family in insects and describe the role of one member, LITAF-like 3 (*LL3*), in anti-*Plasmodium* immunity in the mosquito *Anopheles gambiae*. Silencing of *LL3* greatly increases parasite survival. The gene appears to function as a transcription factor that binds to specific regions of the *SRPN6* promoter, a known anti-*Plasmodium* gene, and modulates its transcript abundance. In summary, *LL3* appears to be a novel component of the mosquito innate immune response.

## Introduction

In virtually all species, the innate immune system is responsible for the primary response against pathogens. Unlike adaptive immunity, the innate immune response does not confer long-lasting protection but instead, relies on the recognition of pathogen–associated molecular patterns (PAMPS). Following recognition, cell-mediated responses eliminate the pathogen. In vertebrates, these responses involve inflammation and the recruitment of specialized cells to the site of infection via the production of effector molecules such as cytokines. As an important mediator of immune regulation, the cytokine tumor necrosis factor-alpha (*TNF-α*) has a variety of functions including apoptotic cell death, inflammation, immune signaling via NF-κB, and cellular proliferation/differentiation [1]. With such pleiotropic functions, it is critical that the expression of *TNF-α* be tightly regulated. Several components have been identified that are involved in the regulation of *TNF-α*, including the LPS-induced TNF-α factor (*LITAF*) [2], [3]. Identified as a transcription factor, LITAF binds to the *TNF-α* promoter in response to bacterial LPS stimulation to influence the expression of *TNF-α* [3], as well as additional LPS-induced cytokines [4].

The components of the mosquito innate immune system are of important biological relevance but are incompletely characterized. Much of our knowledge of the mosquito innate immune system is based on homologous innate immune pathways first described in *Drosophila*. Although evolutionarily distant from the well-characterized vertebrate TLR innate immune pathways, analogous mosquito *Toll* and *IMD* pathways drive the nuclear translocation of NF-κB-like transcription factors to provide defense against invading pathogens via expression of anti-microbial peptides. The activation of the *Toll*, *IMD*, and *JAK-STAT* pathways [5]–[7], have been shown to limit the success of the malaria parasite *Plasmodium*. Several effector genes have been identified that influence *Plasmodium* development in the mosquito [8], yet many questions remain as to how the mosquito immune response recognizes and destroys invading pathogens.

Here we report on the first identification of a *LITAF*-like gene in insects and investigate its role in the mosquito immune response to *Plasmodium*. Similar to vertebrate *LITAF*, *LL3* seems to act as a transcription factor involved in the regulation of the mosquito immune response, as evidenced by its direct effects on the expression of *SRPN6*, a known anti-*Plasmodium* effector gene [9], [10]. These findings demonstrate for the first time the role of a *LITAF*-like gene in insects and suggest that *LL3* is an integral component of the mosquito immune response to limit *Plasmodium* infection.

## Results

### The *An. gambiae* LITAF-like gene family

An expressed sequence tag (EST) corresponding to the annotated gene AGAP009053 in *An. gambiae* was originally identified using a subtractive hybridization cDNA library enriched for mosquito genes following *Plasmodium* infection [11]. The predicted protein product of 82 amino acids, shares sequence similarity to *LITAF*, a transcription factor involved in the activation of *TNF-α* and other cytokines in vertebrate organisms [2], [4]. Furthermore, BLAST analysis revealed six highly conserved *LITAF*-like sequences in the *An. gambiae* genome. Phylogenetic analysis of genes encoding LITAF domain-containing proteins across taxa revealed an expansion within dipteran insects, likely due to an ancient gene duplication event, in contrast to mammals and other invertebrates that contain a single *LITAF* gene (Figure S1A). Each of the six identified LITAF-like proteins in *An. gambiae* have direct orthologues in other mosquito species.

RT-PCR was used to examine the expression pattern of the *An. gambiae LITAF*-like genes in response to *Plasmodium* infection (Figure S1B). Four of the genes, all located within an approximate 50 kb stretch on chromosome 3R, produced specific PCR products and were named *LITAF*-like 1–4. Expression of AGAP009053, or *LITAF*-like 3 (*LL3*), was strongly induced in the midgut of *P. berghei*-infected mosquitoes suggesting that *LL3* is involved in the immune response against *P. berghei* parasites.

### Expression of *LL3* following *Plasmodium* infection

**Figure 1:**
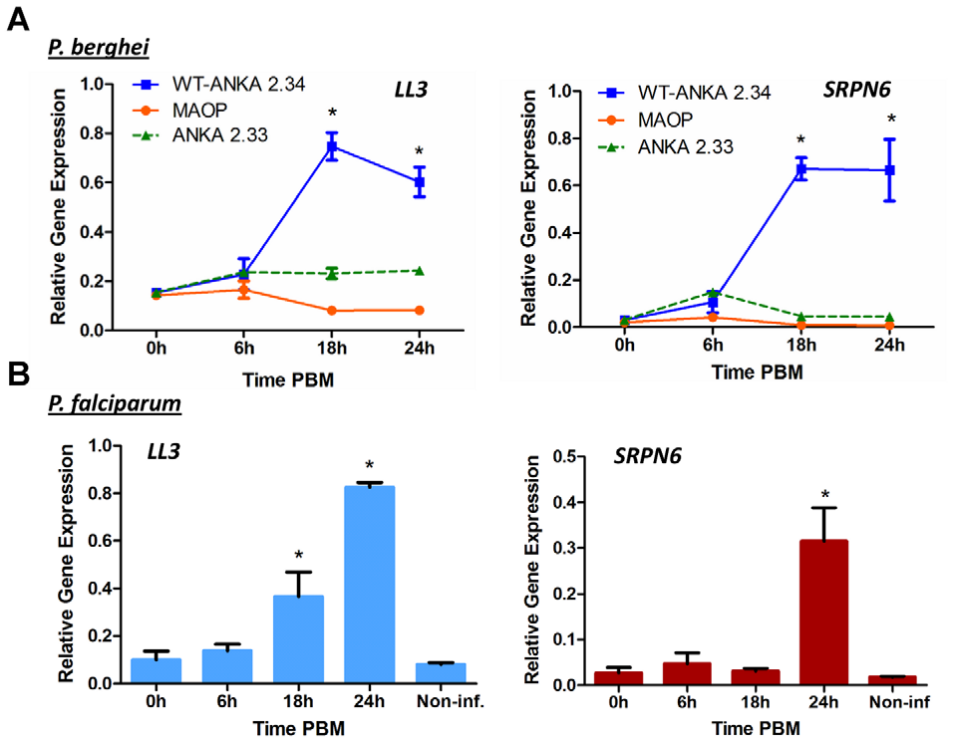
Expression of LL3 in response to *Plasmodium* infection. (A) *An. gambiae* mosquitoes were fed on mice infected with the non-gametocyte producing *P. berghei* ANKA 2.33 strain, with the non-invasive *P. berghei* MAOP mutant, or with the wild type *P. berghei* ANKA 2.34 strain (WT). Midguts were dissected from sugar-fed (0 h) mosquitoes or at different time points following blood meal. LL3 and SRPN6 mRNA abundance was measured by qRT-PCR and normalized to rpS7 (relative gene expression). Data from three independent biological experiments were pooled and analyzed by two-way ANOVA and asterisks denote significant differences (*P*<0.05) when compared to sugar-fed samples using a Dunnet's post-test. (B) LL3 and SPRN6 mRNA abundance was measured by qRT-PCR in midguts from mosquitoes that were sugar-fed (0 h), at different times after a *P. falciparum*-infected blood meal or at 24 h after a non-infectious blood meal (Non-inf). Data from two independent biological experiments were pooled and analyzed by a one-way ANOVA. Asterisks denote significant differences (*P*<0.05) when compared to sugar-fed samples using a Dunnet's post-test.

To more closely characterize the role of *LL3* in the mosquito response to *Plasmodium* infection, qRT-PCR was used to quantify the changes of gene expression in response to a *Plasmodium*-infected blood meal. At the onset of *P. berghei* ookinete midgut invasion (∼18 hours), *LL3* expression is significantly increased and remains high (Figure 1A). Parasites unable to sexually differentiate (ANKA 2.33) or MAOP mutant parasites that produce ookinetes that attach but are unable to invade the midgut [12] fail to induce a response, suggesting that the expression of *LL3* is triggered by the physical invasion of the mosquito midgut. Related experiments with *P. falciparum* show that *LL3* is also induced within a similar time frame (Figure 1B). For both species, a similar pattern of *SRPN6* (AGAP009212) expression was detected, raising the possibility that a common mechanism may regulate the expression of both genes.

### LL3 expression following ookinete invasion

**Figure 2:**
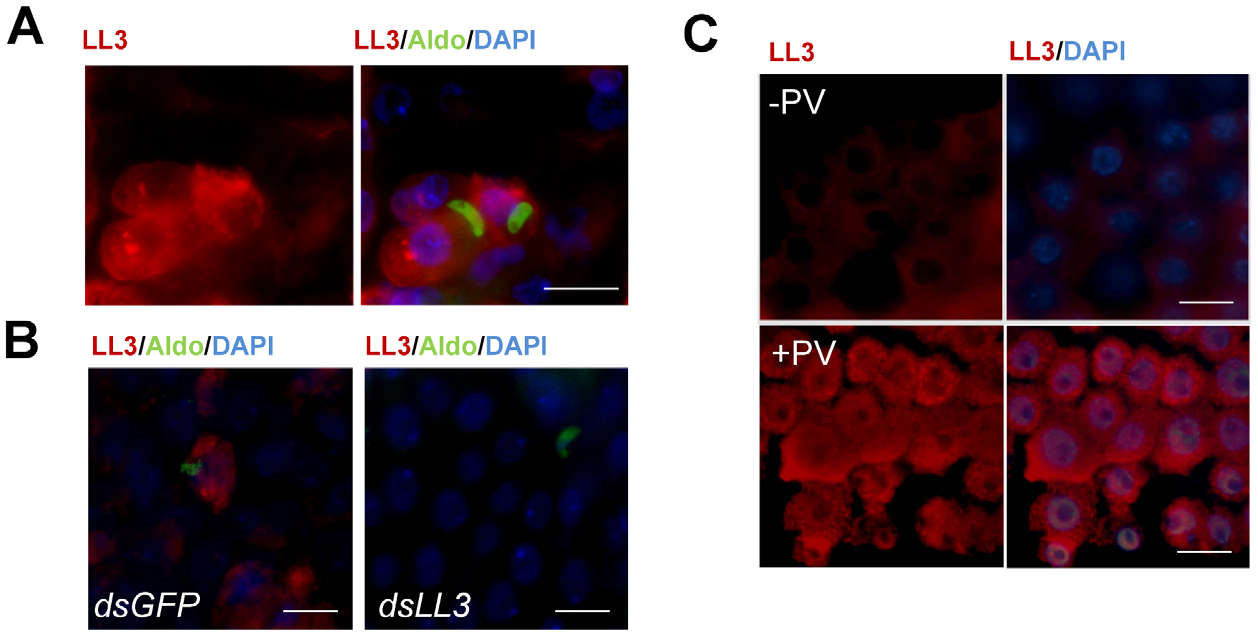
Immunofluorescence localization of LL3 in the mosquito midgut. To determine the localization of LL3 following *P. berghei* infection, midgut sheets were prepared 20 h PBM and visualized using a peptide-derived LL3 antibody. (A) Expression of LL3 (red) was detected in close proximity to invading ookinetes. Images are displayed as LL3 alone (left panel) or as a merged image (right panel) with ookinetes detected by an α-aldolase antibody (green) and DAPI staining (blue) to denote nuclei. (B) dsRNA-mediated silencing of LL3 (dsLL3) correlates with a dramatic reduction in the LL3 signal in comparison with dsGFP controls. Colors of the signals are as indicated on top of each panel. (C) Localization of LL3 following pervanadate treatment. LL3 protein staining was measured in control midgut sheets (−PV) or in midguts following treatment with pervanadate (+PV). Images are displayed as LL3 alone or as a merged image with DAPI staining as indicated on the top of each panel. All images are representative of multiple biological replicates. Scale bars denote 20 microns in all images.

LL3 induction following *P. berghei* infection was evaluated by immunofluorescence assays using a peptide-derived LL3 antibody. LL3 signal above background was detected only in midgut cells in close proximity to ookinetes (Figure 2A), suggesting that LL3 expression is induced by parasite invasion. This response was further confirmed by immunofluorescence assays following the dsRNA-mediated silencing of *GFP* (control) or *LL3* to confirm the specificity of the LL3 signal (Figure 2B). These results are consistent with the weak fluorescence obtained when mosquitoes were fed with the invasion-deficient MAOP mutant parasites suggesting that LL3 protein expression correlates directly with *LL3* transcript abundance (Figure S2). Moreover, the overall response of LL3 to parasite invasion resembles that previously described for SRPN6 [9].

In cells that strongly express LL3, fluorescence is detected in both the cytoplasm and nucleus (Figure 2A and S2). Although expression is primarily localized to the cytoplasm, a small proportion of the signal is detected in the nucleus that may be sufficient for transcriptional activation. While the mechanisms mediating LL3 nuclear translocation remain undefined, this may be regulated by post-translational modifications similar to LITAF in mammals [4], and as is the case for REL1, REL2 and STAT1 in *An. gambiae* [13]. No signal was obtained following the incubation with pre-immune sera (Figure S2).

### LL3 is induced by pervanadate

Previous experiments have shown in mammals that LITAF translocation to the nucleus in response to LPS treatment is phosphorylation dependent [4]. As a result, we wanted to determine the role of phosphorylation on the translocation of LL3 to the nucleus. However, due to the variability in the kinetics of *Plasmodium* midgut invasion and the often transient nature of transcription factor activation, we employed an alternative approach through pervanadate treatment as previously done to examine STAT translocation [14]. As a mixture of sodium orthovanadate and hydrogen peroxide, pervanadate induces oxidative stress mimicking the environment of midgut cells following *Plasmodium* invasion and acts as a potent phosphatase inhibitor. To investigate the LL3 response to pervanadate, immunofluorescence assays were performed to determine LL3 activation and nuclear translocation (Figure 2C).

In control mosquitoes (−PV), only a weak fluorescence signal was detected with LL3 immune sera. Upon pervanadate treatment (+PV), LL3 was strongly expressed in all cells and appears to be localized in both the nucleus and the cytoplasm (Figure 2C). This suggests that LL3 expression is quickly induced in response to pervanadate treatment likely due to cell stress caused by increased reactive oxygen, to its strong phosphatase inhibitor activity, or a combination of the two. While this does not directly link nuclear translocation to LL3 phosphorylation, it does provide preliminary results to further explore the basis of LL3 activation.

### Silencing of *LL3* substantially increases oocyst numbers

**Figure 3:**
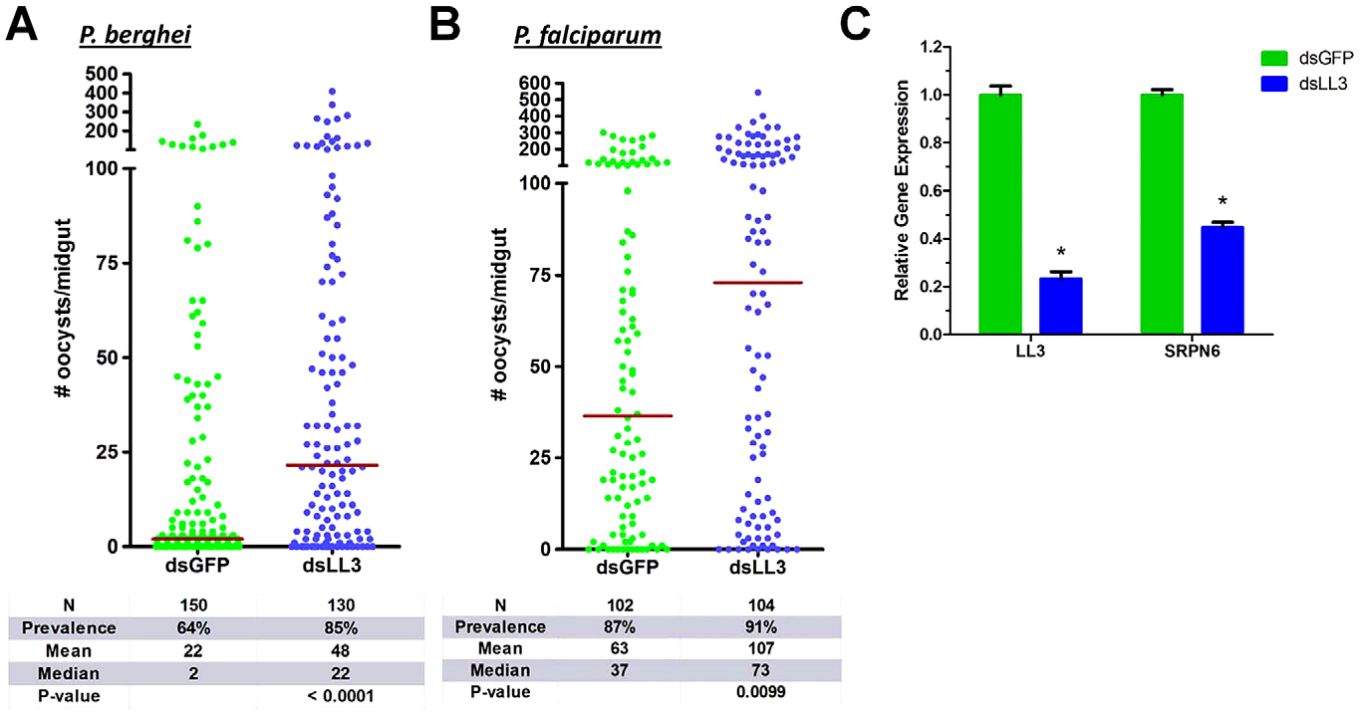
*Plasmodium* oocyst intensity increases in LL3 knockdown mosquitoes. GFP (control) or LL3 dsRNA was injected into adult female mosquitoes and two days later, surviving mosquitoes were given an infectious blood meal containing *P. berghei* (A) or *P. falciparum* (B) parasites. Oocyst numbers were determined respectively at 10 or 7 days following the blood meal. Data from six (*P. berghei*) or three (*P. falciparum*) independent experiments were pooled and analyzed by the Mann-Whitney test. The horizontal bars denote the median value for each experimental group. N: number of midguts assayed; Prevalence: percent of midguts that had at least one oocyst. (C) Efficiency of LL3 knockdown and its effect on SRPN6 mRNA abundance were assessed in midguts of *P. berghei*-infected mosquitoes 24 h PBM. The relative gene expression represents transcript abundance normalized to the GFP control across four separate experiments. Data were compared with the Student's t-test to determine differences in LL3 and SRPN6 expression between GFP control and LL3 knockdown samples. Asterisks denote a *P* value of <0.05.

We used RNAi-mediated gene silencing to determine whether *LL3* plays a role in the mosquito response to *Plasmodium* infection. Silencing of *LL3* led to a substantial increase in *P. berghei* oocyst numbers and infection prevalence when compared to ds*GFP* controls (Figure 3A). Infection by *P. falciparum* parasites was similarly affected as the *LL3* knockdown mosquitoes displayed double the oocyst load when compared to controls (Figure 3B). A small, but non-significant increase in the *P. falciparum* infection prevalence was detected, despite the high intensity of the controls. Previous reports have suggested that the immune responses of the mosquito to *P. berghei* and *P. falciparum* are quite divergent [15]. Several molecules that have been implicated in anti-*Plasmodium* defenses only function against a specific parasite species [8]. However, the dsRNA-mediated silencing of *LL3* results in a significant increase in the number of developing oocysts for both rodent and human parasite species, suggesting that *LL3* is a universal component of the mosquito anti-*Plasmodium* response. The presumed role of LL3 as a transcription factor suggests that it regulates the immune response at the transcriptional level.

*LL3* knockdown efficiency was verified by qRT-PCR and resulted in an approximate 80% reduction in mRNA abundance (Figure 3C). To determine the specificity of *LL3* silencing, the expression of the other *LITAF*-like genes was monitored by RT-PCR (Figure S3). Only *LL4* displayed a slight decrease in expression, but further experiments are needed to determine if this is a downstream target of LL3 activation. Significantly, *LL3* knockdown also resulted in a considerable decrease in *SRPN6* expression (Figure 3C), a known inhibitor of *Plasmodium* development [9], [10].

### Identification of LL3 binding sites

**Figure 4:**
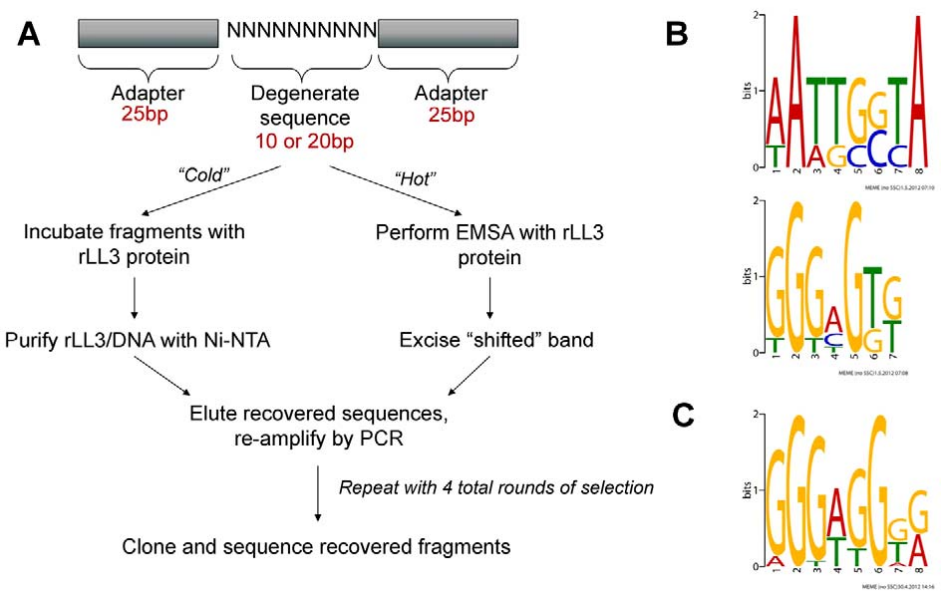
PCR-assisted DNA-binding site selection reveals consensus LL3 DNA-binding motifs. (A) Experimental outline of the two methods [“cold” (non-radioactive) or “hot” (radioactive)] used to obtain consensus DNA binding sites for rLL3 by PCR-assisted DNA-binding site selection. Consensus motifs obtained from the “cold” method using a 10 bp degenerate sequence (B) or from the 20 bp degenerate sequences recovered using the “hot” method (C) are shown to the right. All recovered sequences used as input to generate the consensus motifs are listed in Table S2 and all motifs generated by the MEME program are displayed in Figure S4. EMSA: Electrophoretic Mobility-Shift Assay.

Based on the characterization of LITAF as a transcription factor in other organisms, we examined the possibility that LL3 may also play a similar role in *Anopheles* and bind DNA. We used two different PCR-assisted DNA-binding site selection assays to identify DNA fragments able to bind to recombinant LL3 (Figure 4A). Following four rounds of selection for each method, the recovered sequences (Table S2) were then used as input for MEME analysis [16] to identify putative LL3-DNA binding motifs (Figure S4). Both methods produced multiple putative motifs.

**Figure 5:**
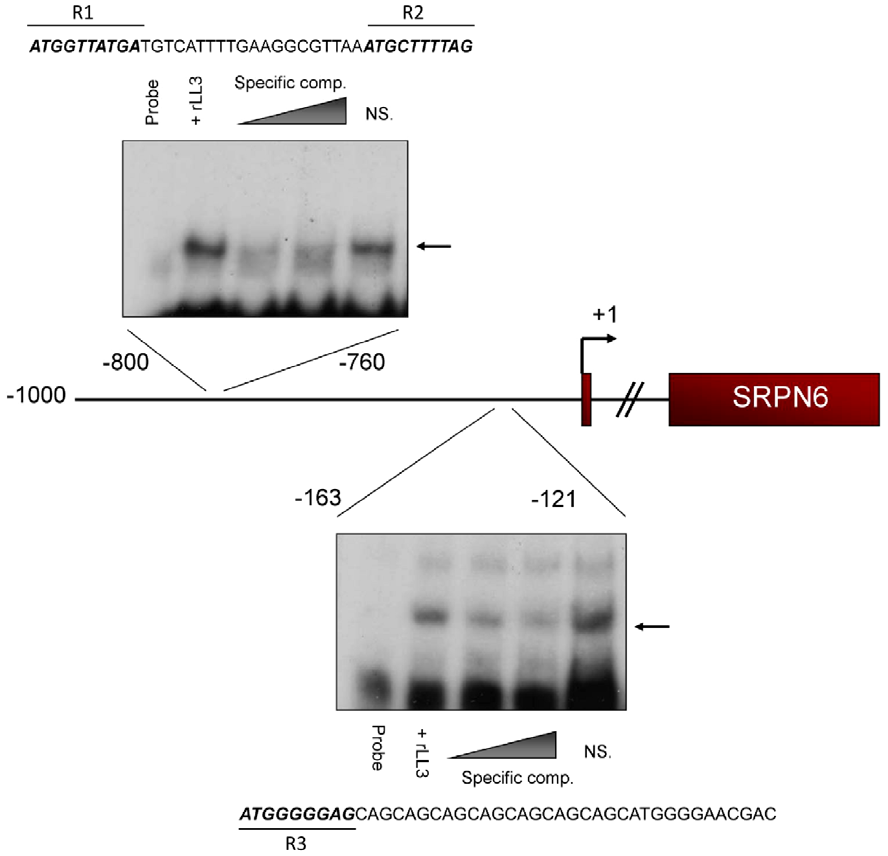
rLL3 binds to specific regions of the SRPN6 promoter. Experiments identified that recombinant LL3 protein interacts with two ∼40 bp regions within the putative SRPN6 promoter (Figs. S5 and S6). Gel shift assays including competition with specific and non-specific (NS) competitors are illustrated for the two regions, with the respective sequences provided above or below. The nucleotides identified by mutational analysis (Figure S6) as being critical for LL3-DNA interactions are in bold italics and labeled as R1 through R3.

**Figure 6:**
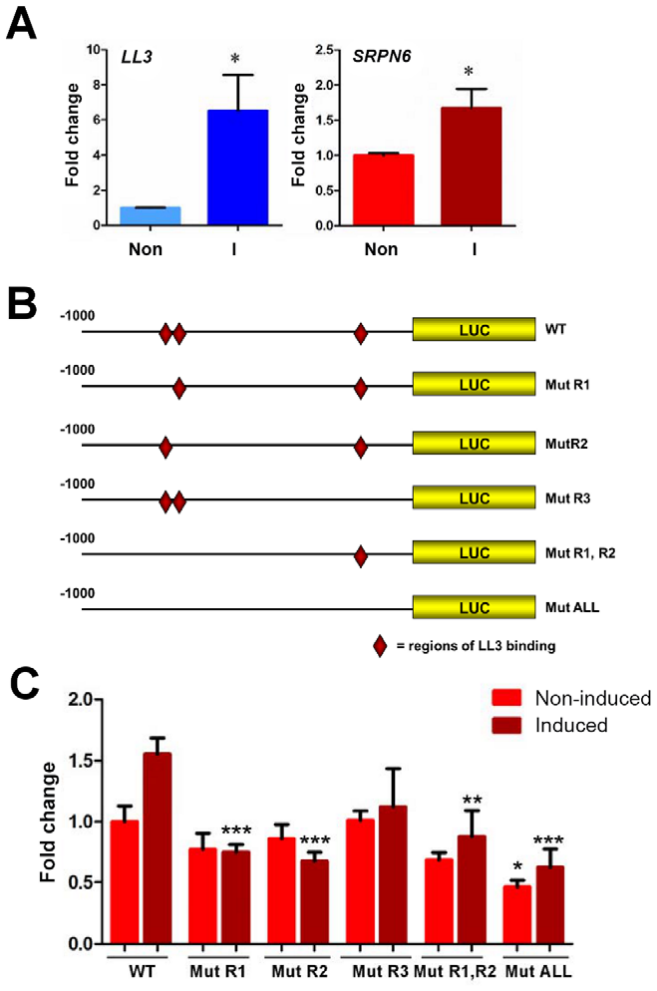
Luciferase expression in a mosquito cell line suggests the involvement of LL3 in the regulation of SRPN6 expression. Expression of LL3 and SRPN6 mRNAs in response to heat-killed *E. cloacae* was investigated in hemocyte-like Sua5B cells (A). LL3 and SRPN6 mRNA abundance was determined by qRT-PCR in cells that were non-induced (non) or after exposure to *E. cloacae* for 6 h (I). Transcript abundance was normalized to that of rpS7 in two independent biological samples and analyzed by the Student's t-test for significance. Asterisks denote significant changes upon bacterial induction (*P*<0.05). The constructs outlined in (B) were used to assess the ability of LL3 to modulate luciferase expression from a SRPN6 promoter. Red diamonds indicate the locations of LL3-binding sites in the wild type promoter (wt) and their presence/absence in each of the mutated promoter constructs. Individual mutants (R1, R2, or R3) correspond to those sites shown in Figure 5, and were combined to create double (R1,R2) or triple mutants (ALL). (C) Each construct was transfected into Sua5B cells and luciferase expression was measured under basal conditions (non-induced) or upon induction with heat-killed *E. cloacae* (induced). Expression was normalized to that of the non-induced wild type SRPN6 promoter in triplicate experiments. The values of two biological repeat experiments were pooled. Asterisks denote significant changes when compared to the wild type construct for each treatment (* = P<0.05, ** = P<0.01, or *** P<0.001) as determined by a Two-way ANOVA and Bonferroni post-test.

For both methods, the most frequently recovered consensus sequence was a GGG[A/T]G motif (Figures 4B, 4C and S4), providing validation of our approach and suggesting that this is a high affinity DNA-binding site for LL3. This motif also shares a striking resemblance to the CTCCC motif (reverse complement of the LL3 motif) described for LITAF on the *TNF-α* promoter [3]. An additional, highly degenerate motif was also identified within the affinity-based enrichment (Figure 4B). The two motifs were chosen from those identified by MEME analysis (Figure S4) based on their presence in the *SRPN6* promoter and likely role in *SRPN6* regulation (Figures 5 and 6).

### LL3 binds to specific regions of the *SRPN6* promoter

Having obtained evidence that LL3 binds to specific DNA sequences, we next investigated whether this putative transcription factor is capable of binding to DNA in the *SRPN6* promoter. LL3 recognition of *SRPN6* promoter sequences would suggest that *SRPN6* expression is directly affected by *LL3* expression (Figure 3C). One kilobase of the putative *SRPN6* promoter was initially divided into five equal fragments and examined by electrophoretic mobility shift assay (EMSA). Two positive fragments were further subdivided to finely map LL3 binding sites. Specific LL3 binding was detected to the −800 to −761, and −163 to −121 regions (Figure S5). These ∼40 bp regions were then examined by mutational analysis to further narrow the sequences of LL3-DNA interactions (Figure S6). The results of these experiments are summarized in Figure 5.

Two 10 bp regions within the −800 to −761 fragment were identified as being critical for LL3-DNA interactions (Figure S6). These sequences from −800 to −791 (R1) and −770 to −761 (R2) are highly similar and closely resemble the consensus sequence described in Figure 4B (top). Within the −163 to −121 fragment, binding was attributed to a 10 bp region (R3) in which a GGGAG motif was identified similar to that detected in Figure 4B (bottom) and Figure 4C. In addition, each of the identified regions share the first 3 bp (ATG), but it is unclear how these residues influence LL3 binding (Figure 5). This close correlation of the LL3 DNA binding motifs identified by two independent methods (PCR-assisted selection and EMSA) strongly reinforces the validity of these results and the likelihood that LL3 directly regulates *SRPN6* through interactions with its regulatory regions.

### *LL3* is induced in hemocyte-like Sua5B cells in response to bacteria

Taking advantage of the availability of immunoresponsive cell lines for *An. gambiae*, we examined the transcriptional response of *LL3* to heat-killed *Enterobacter cloacae* in the hemocyte-like Sua5B cell line. When challenged with heat-killed bacteria, *LL3* expression was significantly increased when normalized to basal levels of transcript in the non-induced cDNA sample (Figure 6A). In addition, the levels of *SRPN6* transcript are also significantly increased, despite being expressed at much higher levels of basal transcription (data not shown).

### *SRPN6* expression is regulated by LL3 binding to the *SRPN6* promoter

In view of the reduced *SRPN6* mRNA abundance when *LL3* is silenced (Figure 3C) and the detection of LL3 DNA-binding motifs in the *SRPN6* promoter (Figure 5), we further investigated the possibility that LL3 directly contributes to the regulation of *SRPN6* expression. With this aim, we built constructs that placed firefly luciferase coding sequence under the control of the *SRPN6* promoter and quantified its expression in Sua5B cells where *LL3* can be induced by the addition of bacteria (Figure 6A). Plasmids carrying the wild type *SRPN6* promoter or promoters containing mutations in the three putative LL3 DNA-binding motifs (Figure 6B) were used for these experiments.

Luciferase expression was measured in naïve cells or following induction with heat-killed *E. cloacae*. A moderate but non-significant increase of luciferase expression after bacteria induction was detected when the gene was driven by the wild type promoter (Figure 6C), similar to the induction levels for the endogenous *SRPN6* transcript (Figure 6A). Upon bacteria induction, a significant decrease in luciferase expression was detected in the promoter constructs containing one or multiple mutations of the LL3 binding sites, except for the [−162 to −153] (R3) mutation (Figure 6C). In non-induced samples, only the expression construct containing mutations of all three of the LL3 binding sites (ALL mutant; [−800 to −790], [−770 to −760], and [−162 to −153]) resulted in a significant decrease, while the other constructs displayed only marginal differences in basal luciferase expression (Figure 6C). Taken together, it appears that the sites −800 to −791 (R1) and −770 to −760 (R2) play an important role in the transcription and induction of SRPN6, while the site from −162 to −153 (R3) may play a more cooperative role as evidenced by the significant decrease in luciferase expression in the triple mutant construct.

Given the complexity of the *SRPN6* gene (it contains an approximate 3.5 kb intron following the start codon), it is possible that the entire transcriptional machinery was not present in the luciferase constructs. However, the changes in expression upon bacterial challenge were remarkably similar to those of the endogenous *SRPN6* transcript (Figure 6A). In summary, LL3 binding sites seem to be more important for activation of *SRPN6* expression (and perhaps of other genes involved in host defense mechanisms) rather than maintenance of basal expression in cell culture. It is also important to note that the immune response is complex and other factors, in addition to LL3, may also play a role in *SRPN6* gene regulation.

## Discussion

Ookinete invasion of the mosquito midgut represents a critical bottleneck in *Plasmodium* development. To ensure its transmission, the parasite must overcome large parasite losses to reach the basal lamina and evade components of the mosquito hemolymph as it transitions to a mature oocyst [17], [18]. Recent advances have increased our understanding of how the development of *Plasmodium* parasites is restricted in its mosquito host, but our understanding of these mechanisms is incomplete. This report investigates for the first time, possible roles played by a *LITAF*-like transcription factor in the mosquito anti-*Plasmodium* response.

Originally identified from a *P. berghei*-infected midgut subtraction library [11], our observations demonstrate that LL3 expression is induced in response to the physical disruption of the midgut epithelium as a result of *P. berghei* and *P. falciparum* ookinete invasion. These results are consistent with previous gene expression analysis for *LL3* [19], and are remarkably similar to the patterns of *SRPN6* expression identified in previous experiments [9] and in this report. Consistent with these results, immunolocalization experiments imply that LL3 expression occurs in cells of the midgut epithelium in close proximity to invading ookinetes. These LL3-positive cell clusters are similar to those previously described for *SRPN6* [9], and other markers of invaded cells [20]–[22], suggesting that LL3 is expressed as a result of ookinete invasion.

During the invasion process, ookinetes traverse multiple cells before reaching the basal lamina where they begin the transition to an oocyst [20], [21]. Meanwhile, the invaded cells undergo a series of morphological and molecular changes that lead to apoptosis and their ultimate removal into the midgut lumen [21], [22]. These damaged cells are marked by elevated levels of nitric oxide synthase (NOS), an enzyme involved in the production of nitric oxide, that create a highly toxic environment in which the ookinete must reach the basal lamina to survive according to the “time bomb” theory of invasion [21], [22]. As a result, the rate at which the ookinete crosses the cell could greatly determine invasion success [21], [22]. Recent work has identified that together with NOS, enzymes that mediate protein nitration within invaded cells are required to effectively label ookinetes for recognition and TEP1-mediated lysis [23]. With a presumed role as a transcription factor, LL3 may be connected to these events by promoting a transcriptional program that leads to parasite recognition by the mosquito complement system, explaining the increased parasite numbers in LL3-silenced mosquitoes. Alternatively, the increased parasite survival associated with *LL3*-silencing may be attributed to a “late-phase” phenotype as described for components of the STAT pathway [7]. The identification of the mechanisms of LL3 anti-*Plasmodium* immunity will be a major focus of future experiments.

Through the use of PCR-assisted DNA-binding site selection assays, we identified several DNA fragments that are recognized by LL3. Although there is inherent noise within the experimental system, MEME analysis identified putative motifs that were independently replicated, providing validation of this approach. From both assays, the predominant motif identified was a GGG[A/T]G consensus sequence. Interestingly, this is the reverse complement to the CTCCC motif that LITAF recognizes on the *TNF-α* promoter and suggests that LL3 binding site recognition is conserved in mosquitoes. Presumably, LL3 influences the regulation of a large repertoire of genes involved in the mosquito innate immune response through interactions with the GGG[A/T]G sequence or other predicted motifs. Bioinformatics approaches to identify putative downstream targets of LL3 in mosquitoes have been further complicated by the short length of the GGG[A/T]G motif, resulting in large numbers of candidate target genes that await further validation. In addition, very little is known regarding the downstream targets of mammalian LITAF, thus providing little information to search for orthologous genes under the control of LL3 in mosquitoes. Identifying the genes under LL3 regulatory control remains a priority for future investigation.

From our experiments, it is clear that LL3 has a direct role in at least one previously described component of the mosquito immune response, *SRPN6* [9], [10]. Upon LL3-silencing, we detect a significant decrease in *SRPN6* transcript in *An. gambiae* following *P. berghei* infection, and have identified LL3 recognition elements in the *SRPN6* promoter that directly regulate *SRPN6* expression in cultured cells. Annotated as a predicted serine protease inhibitor or serpin, similar serpin family members have been implicated in the down-regulation of immune pathways in *An. gambiae* through their interaction with a target protease [9], [20], [24], [25]. However, the precise role of SRPN6 in the immune response has yet to be elucidated and is further confounded by the complex phenotype obtained following SRPN6 knockdown in which the developmental success of *P. berghei* varies on the species and strain of the mosquito host [9]. SRPN6-silencing in susceptible lines of *An. gambiae* did not impact infection intensity, but implicate SRPN6 function in parasite clearance [9]. Based upon the large increase in oocyst numbers following LL3 knockdown in *An. gambiae* with *P. berghei* and *P. falciparum*, it is clear that the effects of LL3-silencing resonate well beyond the regulation of SRPN6 in the mosquito anti-*Plasmodium* response.

Taken together, we provide the first description of *LITAF*-like genes in dipteran insects and demonstrate the involvement of at least one member of this class of putative transcription factors as a novel component of the *An. gambiae* innate immune response. Our findings provide an important starting point for further investigation into the mechanisms of LL3 function and the targets under its regulatory control. New questions regarding the identification of signaling pathways involved in LL3 activation will be addressed and efforts will be made to place LL3 in the overall context of mosquito immunity. Based upon its homology to mammalian *LITAF*, one may speculate that LL3 influences the expression of a TNF-α-like molecule or other yet unidentified cytokines involved in the mosquito innate immune response. It will also be interesting to examine if *LL3* or other *LITAF*-like genes also influence *SRPN6* expression in the mosquito salivary glands, where SRPN6 has also been implicated in limiting *Plasmodium* sporozoite invasion [10]. In conclusion, these results provide evidence for a new component of the mosquito response to *Plasmodium* infection. Further work may lead to improved strategies to curtail the transmission of malaria.

## Materials and Methods

### Ethics statement

This project was carried out in accordance with the recommendations of the Guide for the Care and Use of Laboratory Animals of the National Institutes of Health. The animal protocol was approved by the Animal Care and Use Committee of the Johns Hopkins University (protocol number M009H58). Anonymous human blood used for parasite cultures and mosquito feeding was obtained under IRB protocol NA 00019050 approved by the Johns Hopkins School of Public Health Ethics Committee. Informed consent is not applicable.

### Mosquito rearing and *Plasmodium* infections

The colony of *Anopheles gambiae* (Keele strain) was obtained from Drs. Hilary Hurd and Paul Eggleston at Keele University. Mosquitoes were maintained on 10% sucrose at 27°C and 80% relative humidity with a 14/10 h light/dark cycle. For *P. berghei* infections, mosquitoes were fed on anaesthetized Swiss Webster mice infected with ANKA 2.33 (a gametocyte-minus clone), ANKA-GFP [26], or MAOP [12] parasites. *P. falciparum* infections were performed by diluting mature NF54 gametocytes to 0.3% gametocytemia and fed using an artificial membrane feeder. Dissections were performed in 1X PBS, and oocysts counts were performed by midgut dissection at 10 d (*P. berghei*) or 7 d (*P. falciparum*) post-infection, stained with 0.2% mercurochrome and visualized with a compound microscope.

### Real-time quantitative PCR expression analysis

Total RNA was prepared from mosquito cell or tissue samples using TRIzol (Invitrogen), and cDNA was prepared using SuperScriptIII (Invitrogen) according to the manufacturer's protocol. Gene expression was analyzed by quantitative real-time PCR using gene-specific primers and Power SYBR Green PCR Master Mix (Applied Biosystems) on a StepOnePlus Real-Time PCR System (Applied Biosystems). PCR was performed using an initial denaturation of 95°C for 5 min, followed by 40 cycles of 95°C for 15 sec, 65°C for 30 sec, and 70°C for 30 sec. Measurements were taken during the 70°C extension at each cycle and a melting curve was used following amplification to confirm product specificity. qPCR results were normalized using *An. gambiae* ribosomal protein S7 as a reference and target gene expression was analyzed according to the 2^−ΔΔCt^ method [27]. Measurements were performed in triplicate and all experiments were replicated with at least two independent biological samples. Gene-specific primers sequences are as follows; rpS7 (F: 5′-ACCACCATCGAACACAAAGTTGACACT-3′ and R: 5′-CTCCGATCTTTCACATTCCAGTAGCAC-3′), LL3 (F: 5′-GTACGCACGAAAGTGAAGCACGAAT-3′ and R: 5′- AATGTTTGTACGAGCCAATGAACGTGT-3′), and SRPN6 (F: 5′-CTCTACTTCAAAGCCAAGTGGAAGACG-3′ and R: 5′-CTGTATCAGGTACATCGTGCTGGTGTC-3′).

### Expression of recombinant LL3 and antibody production

A 243 bp fragment encompassing the LL3 ORF was amplified from *An. gambiae* midgut cDNA using the following primers; F: 5′-CACCACTACCATCATAGTGACGAACCCGC-3′ and R: 5′-ATGTTTGTACGAGCCAATGAACGTGTTGC-3′. Following gel purification with the Qiaex II gel extraction kit (Qiagen), the LL3 PCR fragment was cloned into a pBAD202/D-TOPO vector (Invitrogen) according to product specifications and later sequenced to verify the sequence accuracy of the inserted DNA. The resulting pBAD-LL3 plasmid was transformed into the BL21 strain of *E. coli* for protein expression. Soluble recombinant LL3 was isolated using BugBuster (Novagen), His-purified using Ni-NTA agarose (Qiagen), and eluted with 50 mM NaH_2_PO_4_, 300 mM NaCl_2_, 250 mM imidazole, and 0.05% Tween. Eluted protein samples were diafiltered using Amicon Ultra columns (Millipore) and 1× PBS for buffer exchange. Individual sample preps were aliquoted in small volumes for one-time use and stored at −80°C.

To generate polyclonal sera in mice for use in immunofluorescence experiments, a LL3 KLH-conjugated peptide (TVRTKVKHESTTSTC) was added to an initial 1∶1 mixture of 1X PBS and Complete Freund's adjuvant (Sigma) and immunized by intra-peritoneal injection. Subsequent boosts (four total) were performed every two weeks as above using incomplete Freund's adjuvant (Sigma) and bleeds were performed to monitor the immune response before the final serum was collected by heart puncture.

### Pervanadate treatment and immunofluorescence microscopy

Midguts from non-infected mosquitoes were dissected in 1X PBS and incubated for 20 min in 1X PBS alone, or with pervanadate treatment as previously described [14]. Immunofluorescence assays were performed as previously described for midgut tissues [20], stained with ProLong Gold antifade reagent with DAPI (Invitrogen) and visualized on a Nikon 90i compound fluorescence microscope. Primary antibody dilutions were made as follows: 1∶500 mouse anti-LL3, 1∶1,000 rabbit anti-Pf aldolase [28]. Secondary Texas Red goat anti-mouse (Invitrogen) or Alexa Fluor 488 goat anti-rabbit (Invitrogen) antibodies were used at a 1∶1,000 dilution.

### dsRNA gene silencing

A 467-bp fragment consisting of the LL3 ORF and 3′ UTR was PCR amplified from midgut cDNA using the primers 5′-ATGACTACCATCATAGTGACGAACCC-3′ and 5′-TTACACCATTATTAAATAAATAACACAACTTGAGATG-3′ and subcloned into a pJet1.2 vector using the CloneJet PCR cloning kit (Fermentas). To create a template for dsRNA, T7 promoter sequences were added to existing gene specific primers to amplify T7-PCR product templates for LL3 (F: 5′-TTAATACGACTCACTATAGGGAGAATGACTACCATCATAGTGACGAACCC-3′ and R: 5′- TTAATACGACTCACTATAGGGAGATTACACCATTATTAAATAAATAACACAACTTGAG-3′) and the GFP control (F: 5′- TTAATACGACTCACTATAGGGAGAATGGTGAGCAAGGGCGAGGAGCTGT-3′ and R: 5′- TTAATACGACTCACTATAGGGAGATTACTTGTACAGCTCGTCCATGCC-3′). The resultant T7-PCR templates were PCR purified and concentrated using the DNA Clean and Concentrator (Zymo Research), then used to produce dsRNA using the MEGAscript RNAi kit (Ambion) according to the manufacturer's protocol. dsRNA products were re-suspended at a final concentration of 3 µg/µl in 1X PBS and used for injections as previously described [29]. Two days post-injection, surviving mosquitoes were fed on *P. berghei*- or *P. falciparum*-infected blood and maintained at 19°C or 25°C respectively. The efficiency of dsRNA-mediated silencing was examined by midgut dissection 24 h post-blood meal and subsequently analyzed by qRT-PCR as described above.

### Selection of LL3 binding sites

To select DNA fragments that bind with affinity to rLL3, a PCR-assisted DNA-binding site selection was performed as previously described with slight modification [30], [31]. Using an oligonucleotide with a random 10-bp region (5′- CGCGGATCCTGCAGCTCGAGN_10_GTCGACAAGCTTCTAGAGCA-3′) as a template, PCR was performed for 12 cycles (1 min at 95°C, 1 min at 55°C, 1 min at 72°C) using the following forward (5′-CGCGGATCCTGCAGCTCGAG-3′) and reverse primers (5′-TGCTCTAGAAGCTTGTCGAC-3′) to amplify a double stranded DNA product. “Cold” selection was performed by incubating the PCR template with 10 µg rLL3 in 15 mM HEPES, 25 mM KCl, and 100 µl Ni-NTA agarose (Qiagen) in 1X PBS. The reaction was incubated at 25°C for 30 min then added to a Poly-Prep Chromatography Column (BioRad) and washed with 1X PBS. DNA was eluted from the bound His-tagged rLL3 by the addition of elution buffer (50 mM NaH_2_PO_4_, 300 mM NaCl, 250 mM imidazole, 0.05% Tween) and used for PCR amplification (20 cycles) of the resulting template for the next round of selection.

An alternate “hot” selection, was performed by end-labeling the forward primer with [γ-^32^P] ATP using T4 polynucleotide kinase (New England Biolabs (NEB)) and used to PCR amplify a DNA template containing a random 20-bp region (5′- CGCGGATCCTGCAGCTCGAGN_20_GTCGACAAGCTTCTAGAGCA-3′) as above with the reverse primer (6 cycles). Labeled fragments were purified using Micro Bio-Spin columns (BioRad) and incubated with ∼1 µg of rLL3 protein for 20 min at room temperature in binding buffer [15 mM HEPES, 25 mM KCl, 2 µg BSA, 2 mM DTT, 10% glycerol, and 100 ng M13 reverse primer (Invitrogen) to reduce non-specific binding]. Reaction components were separated on a 7% polyacrylamide/TBE gel at 100 V for ∼90 minutes at 4°C, then dried and exposed to film. “Shifted” complexes were excised and incubated in TE buffer overnight at 25°C and used for PCR amplification (20 cycles) for the next round of selection.

For both “cold” and “hot” methods, a total of 4 rounds of selection were performed before cloning the amplified template into a pJet1.2 vector (Fermentas) for sequencing. The resulting selected DNA fragments were used as input for MEME analysis to generate consensus motifs [16].

### Electrophoretic Mobility Shift Assays (EMSA)

To identify regions of the SRPN6 promoter that are capable of binding rLL3, a 1 kb region of the putative promoter was dissected into five fragments of 200 bp and amplified by PCR using the primers listed in Table S1. Following PCR purification with the DNA Clean and Concentrator kit (Zymo Research), 1 pM of DNA was radiolabeled with [γ-^32^P] ATP using T4 polynucleotide kinase (NEB) according to the manufacturer's protocol and purified with Micro Bio-Spin columns (BioRad). Radiolabeled fragments were incubated in binding buffer [15 mM HEPES, 25 mM KCl, 2 µg BSA, 2 mM DTT, 10% glycerol, 100 ng M13 reverse primer (Invitrogen), and ∼250 ng of poly dA/dT to reduce non-specific binding] and ∼250 ng rLL3 protein for 20 min in the presence or absence of specific (self) or non-specific (rpS7) competitor fragments. EMSA reactions were fractionated on a 7% polyacrylamide/TBE gel at 100 V for ∼90 min at 4°C, then dried and exposed to film.

A more detailed analysis of the 200 bp fragments that demonstrated putative binding was performed by dissecting each fragment into five fragments of ∼40 bp. Forward and reverse-complementary oligonucleotides were synthesized corresponding to each region (Table S1), annealed to form a double-stranded DNA fragment, and radiolabeled with [γ-^32^P] ATP as previously mentioned. Incubation with rLL3 was performed in the presence of specific (self) or non-specific (AgB2t) competitor fragments.

Supershift assays were performed as described above with the exception that DTT was removed from the binding buffer to not interfere with antibody affinity, since DTT is a strong reducing agent. Anti-His, anti-LL3, or mouse pre-immune sera were added to the binding reactions at a 1∶1000 dilution, and incubated for 20 min before gel loading.

### SRPN6 promoter constructs and luciferase assays

To measure regulation conferred by LL3 binding to the SPRN6 promoter, SRPN6 regulatory regions were cloned into a pGL2-control (Promega) luciferase vector as follows. The 130-bp SRPN6 5′UTR was PCR amplified from cDNA with the primers (restriction sites underlined) F: 5′-ATAAGATCTGTCTCGAGAGCGTACACCAGCGTAACGG-3′ and R: 5′-ATAAAGCTTTGTGGAGCATTCAACTCCAACGTTCAAC-3′. Following restriction digestion with *Bgl*II and *Hind*III, the fragment was gel purified using the Gel DNA Recovery kit (Zymo Research) and cloned into the corresponding *Bgl*II and *Hind*III sites within the pGL2-control vector (Promega). Subsequently, 1 kb of the SRPN6 promoter was PCR amplified (using the primers F: 5′- GCAGCCGGTATGGCCGGTTGTGGTTAAATTC-3′ and R: 5′- TGAATGGCTTCGATCGGCGGTGAAAC-3′), and sub-cloned into the pJet1.2 vector (Fermentas). The SRPN6 promoter fragment was then digested with *Bgl*II and ligated into the corresponding *Bgl*II site in the pGL2-SRPN6-5′UTR construct and sequenced to verify the accuracy of the inserted DNA sequence.

Using phosphorylated primers, putative LL3 binding sites within the SRPN6 promoter were disrupted by PCR mutagenesis as follows. Primer pairs targeting the putative sites from −800 to −790 (F: 5′-CCGGCACTAGCTCCaaaaaaaaaaTGTCATTTTGAAGGCGTTAAA-3′, R: 5′-ATGAAAACGATTCTGTTTCAATGTGTTTACGGTGCAGT-3′), −770 to −760 (F: 5′- TTTTGAAGGCGTTAAaaaaaaaaaaAGTGTGTTTAAGCTTCCG-3′, R: 5′-TGACATCATAACCATGGAGCTAGTGCCGGAAGAAAACG-3′), and −162 to −153 (F: 5′-CGTCCAAGCACTCCAaaaaaaaaaaAGCAGCAGCAGCAGCAGCAGC-3′, R: 5′- GTAAAAGTGCAAAATTTGCAATCGCAAATGGCACC-3′) using Phusion polymerase (NEB) and the pGL2-SRPN6 promoter plasmid as a template. Following PCR, the linearized plasmids were ligated and transformed, then sequenced to verify the mutation. Double and triple mutants were sequentially created by a second or third PCR mutagenesis using a mutated −770 to −760 template as outlined above.

Luciferase assays were performed by transfecting a 100∶1 ratio of each respective SRPN6 firefly luciferase promoter construct with a *Renilla* luciferase internal control construct under the *copia* promoter in *Anopheles gambiae* Sua5B cells using a standard Effectene (Qiagen) protocol. Luciferase expression was measured using the Dual Luciferase Reporter Assay System (Promega) in the presence, or absence, of heat-killed *E. cloacae* following the protocol outlined in Gupta *et al.* [7]. Each sample was measured in triplicate and experiments were performed in duplicate.

## Supporting Information

Figure S1 **Characterization of LITAF domain-containing genes.** (A) Coding sequences containing LITAF domains across different taxa were arranged by Clustal W and analyzed using MEGA5 software to construct a maximum-parsimony tree with bootstrapping (n = 1000). Individual protein accession numbers and species names are shown for each sequence, and when available, designated by their gene name. Bootstrap values are displayed next to each node. Within Diptera and in contrast to higher vertebrates, an expansion of genes encoding LITAF domain-containing proteins has occurred. (B) RT-PCR analysis of *An. gambiae* LITAF-like transcripts in dissected midguts and carcasses (whole mosquitoes minus guts) 24 h after feeding on a non-infectious (24 h Non) or on a *P. berghei*-infected (24 h Pb) blood meal. Expression of LL3 was consistently upregulated in midguts following infection with *P. berghei*. Preliminary attempts with gene-specific primers for AGAP002435 (LL5) or AGAP004928 (LL6) did not amplify PCR products (data not shown). SRPN6 expression is shown as a positive control, while rpS7 serves as a loading control. All primer sequences for RT-PCR are listed in Table S1. (−): negative control (complete reaction minus added cDNA). (+): gDNA positive control (reaction primed by genomic DNA).
(TIF)

Figure S2 **Localization of LL3 in invaded mosquito midguts.** Approximately 24 h PBM, mosquito midguts were dissected and visualized by immunofluorescence with an anti-LL3 antibody. Midgut sheets from mosquitoes fed with the non-invasive MAOP mutant parasite give a very weak (or background) signal, and LL3 expression appears to be limited to the cytoplasm. In contrast, LL3 expression is strongly induced by wild type ANKA 2.34 parasites and the protein is detected in both the nucleus and the cytoplasm of the induced midgut cells. No fluorescence was detected in midgut sheets infected with wild type parasites after incubation with the pre-immune sera. Scale bars denote 10 microns.
(TIF)

Figure S3 **Specificity of LL3 dsRNA-mediated knockdown.** To verify the specificity of the LL3 dsRNA knockdown, RT-PCR was performed on midguts of *P. berghei*-infected mosquitoes injected with dsGFP or dsLL3. Using rpS7 as a loading control, gene expression was compared between control and experimental samples for each of the LITAF-like genes in which transcript was detected in Figure S1B. Primers used are listed in Table S1. (−): negative control (complete reaction minus added cDNA); (+): gDNA positive control (reaction primed by genomic DNA).
(TIF)

Figure S4 **LL3 DNA-binding consensus sequences.** Following four cycles of PCR-assisted DNA-binding site selection as outlined in Figure 4A, DNA fragments were cloned and sequenced to identify the nucleotide sequence of DNA bound by LL3. Sequences obtained from the panning experiments (shown in Table S2) were then used as input for MEME analysis. Consensus motifs generated from the 10-bp panning experiment (N = 30) are displayed in (A) and the 20-bp panning experiment (N = 32) displayed in (B) in order of decreasing prevalence. N = number of input sequences used to generate the motifs out of the input total for each experiment. Consensus sequences matching binding data with the SRPN6 promoter (Figure 5) are denoted by red boxes (solid or dashed line) and displayed in Figures 4B and 4C. Identical GGG[A/T]G motifs were recovered from both experimental procedures (solid red boxes).
(TIF)

Figure S5 **EMSA analysis of the SRPN6 promoter.** The SRPN6 promoter was sequentially dissected by use of the Electrophoretic Mobility Shift Assay (EMSA) to identify regions capable of binding to the recombinant LL3 (rLL3) protein. One kilobase of the presumed SRPN6 promoter was divided into five 200-bp fragments that were PCR amplified, radiolabeled, and tested for their ability to bind to the rLL3 protein. The two 200-bp fragments that bound rLL3 were further analyzed under more stringent conditions. Specific binding to the regions −800 to −601 (A), and −200 to −1 (B) was demonstrated through the addition of specific and non-specific competitors and the presence of a super-shifted band in the presence of α-LL3 or α-His antibodies in the absence of DTT (-DTT)(upper panels). Double-stranded oligonucleotides of ∼40 bp each were used to identify the specific regions of rLL3 binding (lower panels). Analysis of the −800 to-601 region demonstrates strong, specific binding to the −800 to −761 fragment (A lower panel). Similar analysis demonstrates strong binding to the region from −163 to −121 within the −200 to −1 fragment (B lower panel). Components of each reaction are shown above each lane. Regions that display specific binding are underlined and denoted with an asterisk. Specific competitors are non-labeled fragments identical in sequence to the radioactive fragment in the assay, while non-specific competition was performed using a 200-bp rpS7 fragment or a 40-bp fragment from the *Anopheles* β2 tubulin gene. All primers used for PCR amplification of the 200-bp fragments or the syntheses of double-stranded oligonucleotides are shown in Table S1.
(TIF)

Figure S6 **Mutational analysis to investigate LL3 target site specificity.** To delineate the critical nucleotides involved in LL3-DNA interactions, the two ∼40 nucleotide regions −800 to −761 (A), and −163 to −121 (B) of the SRPN6 promoter that demonstrated specific rLL3 binding (Fig. S5) were mutated through the conversion of 10 bp stretches to adenosine residues. For each region, standard competition experiments were performed with specific, non-specific and mutated ds DNA fragments. To identify those DNA sequences involved in LL3-DNA interactions, mutated fragments that compete less efficiently than their wild type counterparts behave as non-specific competitors. In contrast, mutations not involved in LL3-DNA interactions have no effect, and compete as effectively as their wild type counterparts. The components of each reaction are shown above each lane, while the sequences of the wild type and mutated competitors are shown below. Regions of specific binding are denoted as regions R1-3.
(TIF)

Table S1 **Primers used for RT-PCR, amplification of SRPN6 promoter fragments, and oligonucleotide probes.**
(XLSX)

Table S2 **Input sequences for the generation of LL3 DNA-binding site consensus motifs.**
(XLSX)
