# Supplementary figures and images for: Regulation of Anti-Plasmodium Immunity by a LITAF-like Transcription Factor in the Malaria Vector Anopheles gambiae
Source: PLoS Pathog. 2012 Oct 18;8(10):e1002965. doi: 10.1371/journal.ppat.1002965 (PMC3475675; doi:10.1371/journal.ppat.1002965)

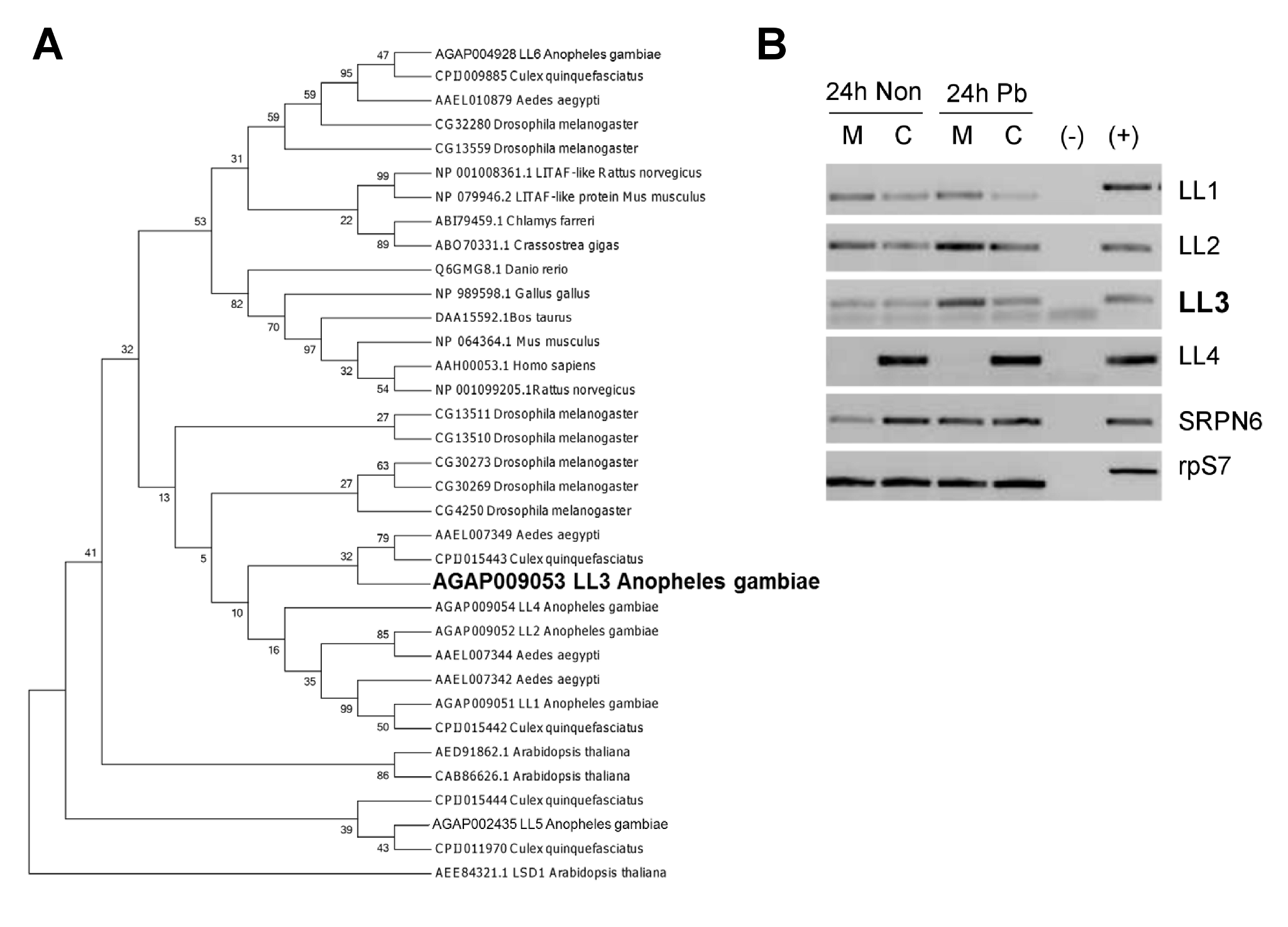

Supplement: Figure S1 — Characterization of LITAF domain-containing genes. (A) Coding sequences containing LITAF domains across different taxa were arranged by Clustal W and analyzed using MEGA5 software to construct a maximum-parsimony tree with bootstrapping (n = 1000). Individual protein accession numbers and species names are shown for each sequence, and when available, designated by their gene name. Bootstrap values are displayed next to each node. Within Diptera and in contrast to higher vertebrates, an expansion of genes encoding LITAF domain-containing proteins has occurred. (B) RT-PCR analysis of An. gambiae LITAF-like transcripts in dissected midguts and carcasses (whole mosquitoes minus guts) 24 h after feeding on a non-infectious (24 h Non) or on a P. berghei-infected (24 h Pb) blood meal. Expression of LL3 was consistently upregulated in midguts following infection with P. berghei. Preliminary attempts with gene-specific primers for AGAP002435 (LL5) or AGAP004928 (LL6) did not amplify PCR products (data not shown). SRPN6 expression is shown as a positive control, while rpS7 serves as a loading control. All primer sequences for RT-PCR are listed in Table S1. (−): negative control (complete reaction minus added cDNA). (+): gDNA positive control (reaction primed by genomic DNA). (TIF) [file ppat.1002965.s001.tif]

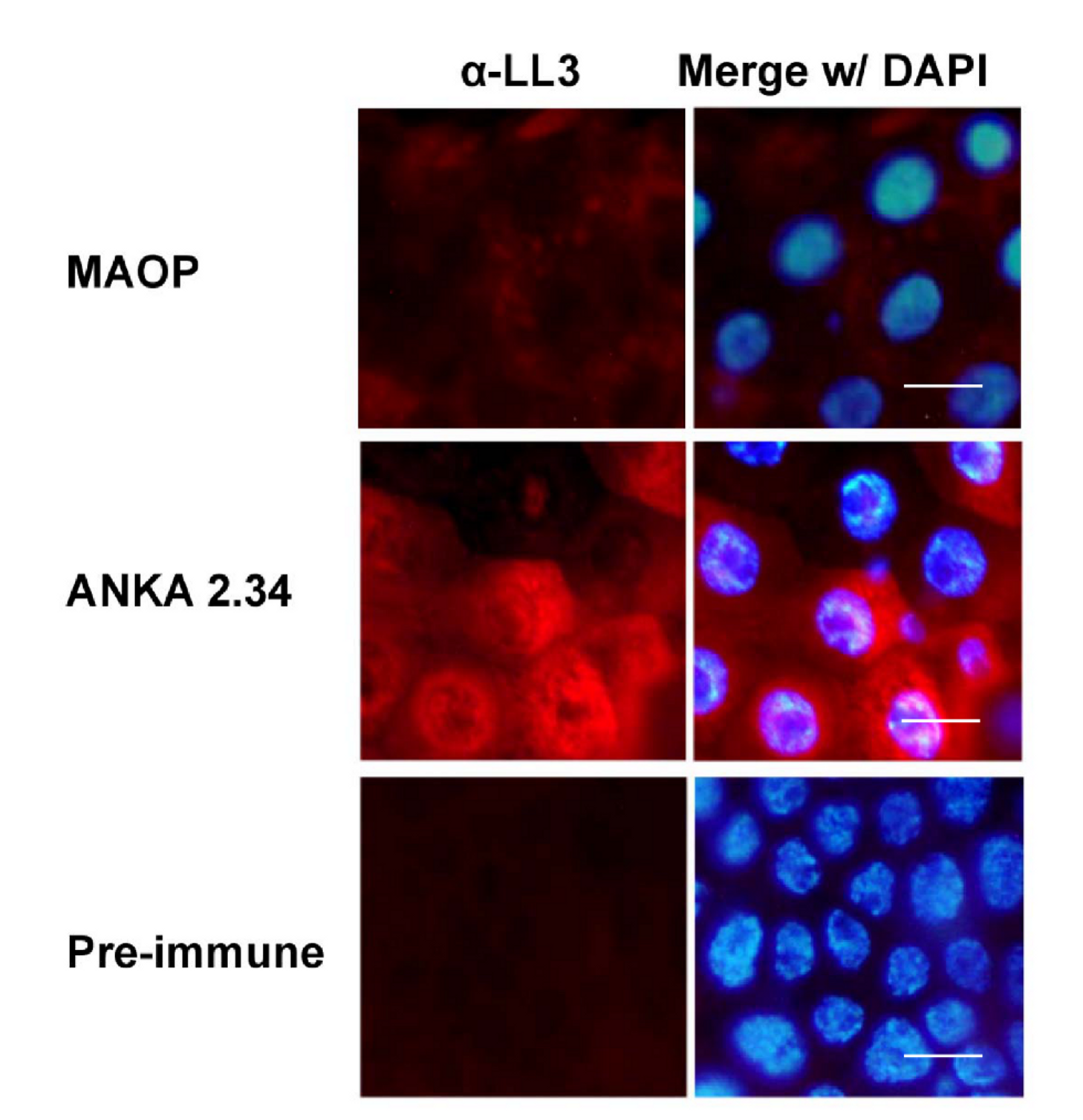

Supplement: Figure S2 — Localization of LL3 in invaded mosquito midguts. Approximately 24 h PBM, mosquito midguts were dissected and visualized by immunofluorescence with an anti-LL3 antibody. Midgut sheets from mosquitoes fed with the non-invasive MAOP mutant parasite give a very weak (or background) signal, and LL3 expression appears to be limited to the cytoplasm. In contrast, LL3 expression is strongly induced by wild type ANKA 2.34 parasites and the protein is detected in both the nucleus and the cytoplasm of the induced midgut cells. No fluorescence was detected in midgut sheets infected with wild type parasites after incubation with the pre-immune sera. Scale bars denote 10 microns. (TIF) [file ppat.1002965.s002.tif]

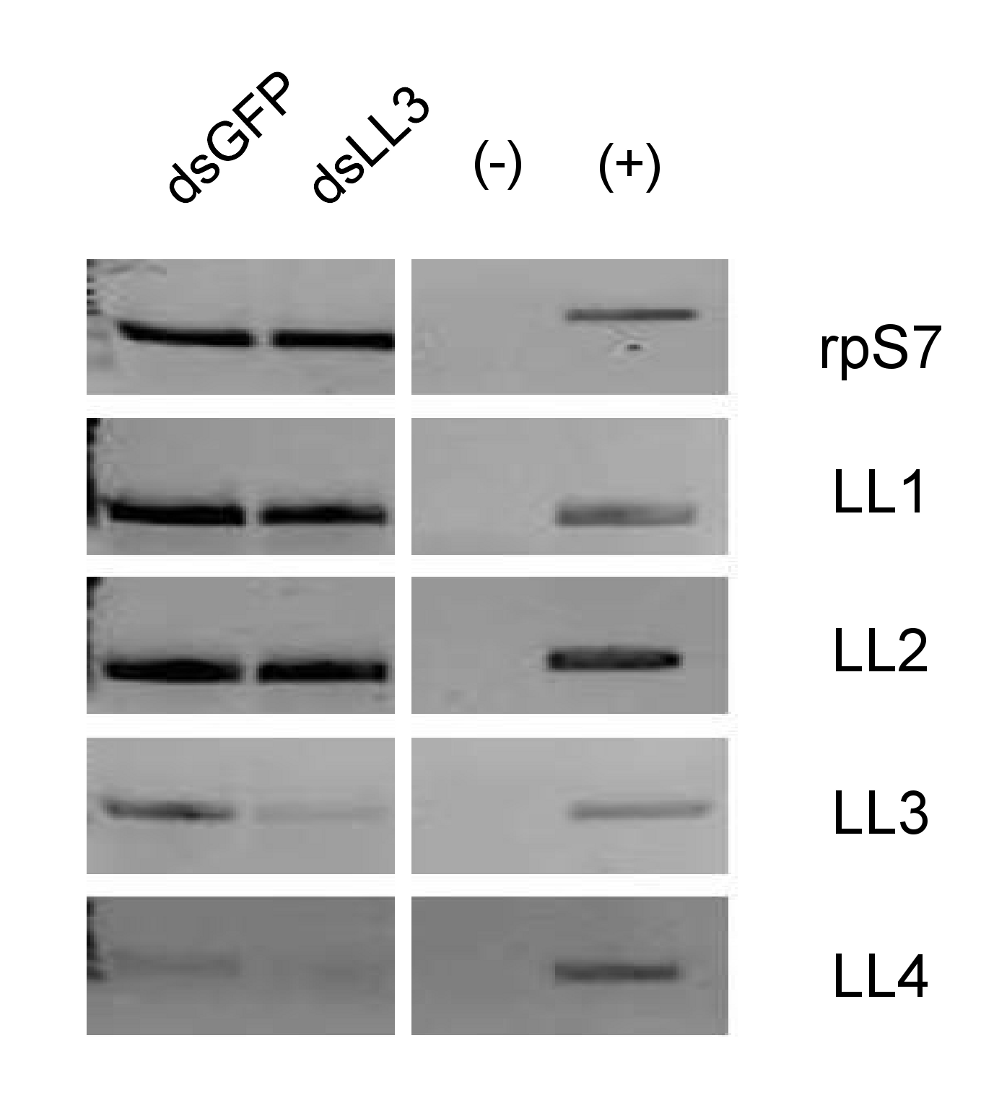

Supplement: Figure S3 — Specificity of LL3 dsRNA-mediated knockdown. To verify the specificity of the LL3 dsRNA knockdown, RT-PCR was performed on midguts of P. berghei-infected mosquitoes injected with dsGFP or dsLL3. Using rpS7 as a loading control, gene expression was compared between control and experimental samples for each of the LITAF-like genes in which transcript was detected in Figure S1B. Primers used are listed in Table S1. (−): negative control (complete reaction minus added cDNA); (+): gDNA positive control (reaction primed by genomic DNA). (TIF) [file ppat.1002965.s003.tif]

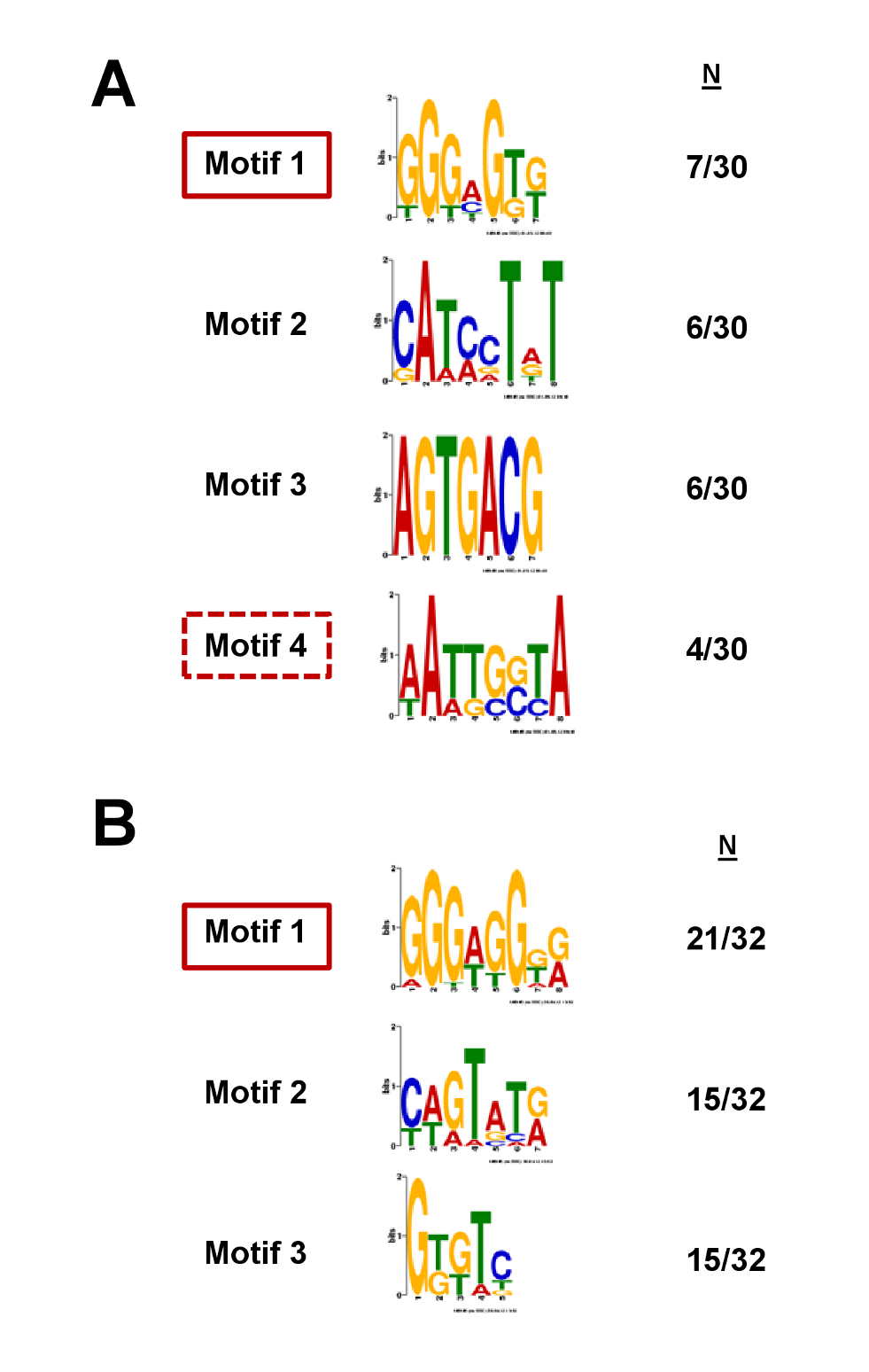

Supplement: Figure S4 — LL3 DNA-binding consensus sequences. Following four cycles of PCR-assisted DNA-binding site selection as outlined in Figure 4A, DNA fragments were cloned and sequenced to identify the nucleotide sequence of DNA bound by LL3. Sequences obtained from the panning experiments (shown in Table S2) were then used as input for MEME analysis. Consensus motifs generated from the 10-bp panning experiment (N = 30) are displayed in (A) and the 20-bp panning experiment (N = 32) displayed in (B) in order of decreasing prevalence. N = number of input sequences used to generate the motifs out of the input total for each experiment. Consensus sequences matching binding data with the SRPN6 promoter (Figure 5) are denoted by red boxes (solid or dashed line) and displayed in Figures 4B and 4C. Identical GGG[A/T]G motifs were recovered from both experimental procedures (solid red boxes). (TIF) [file ppat.1002965.s004.tif]

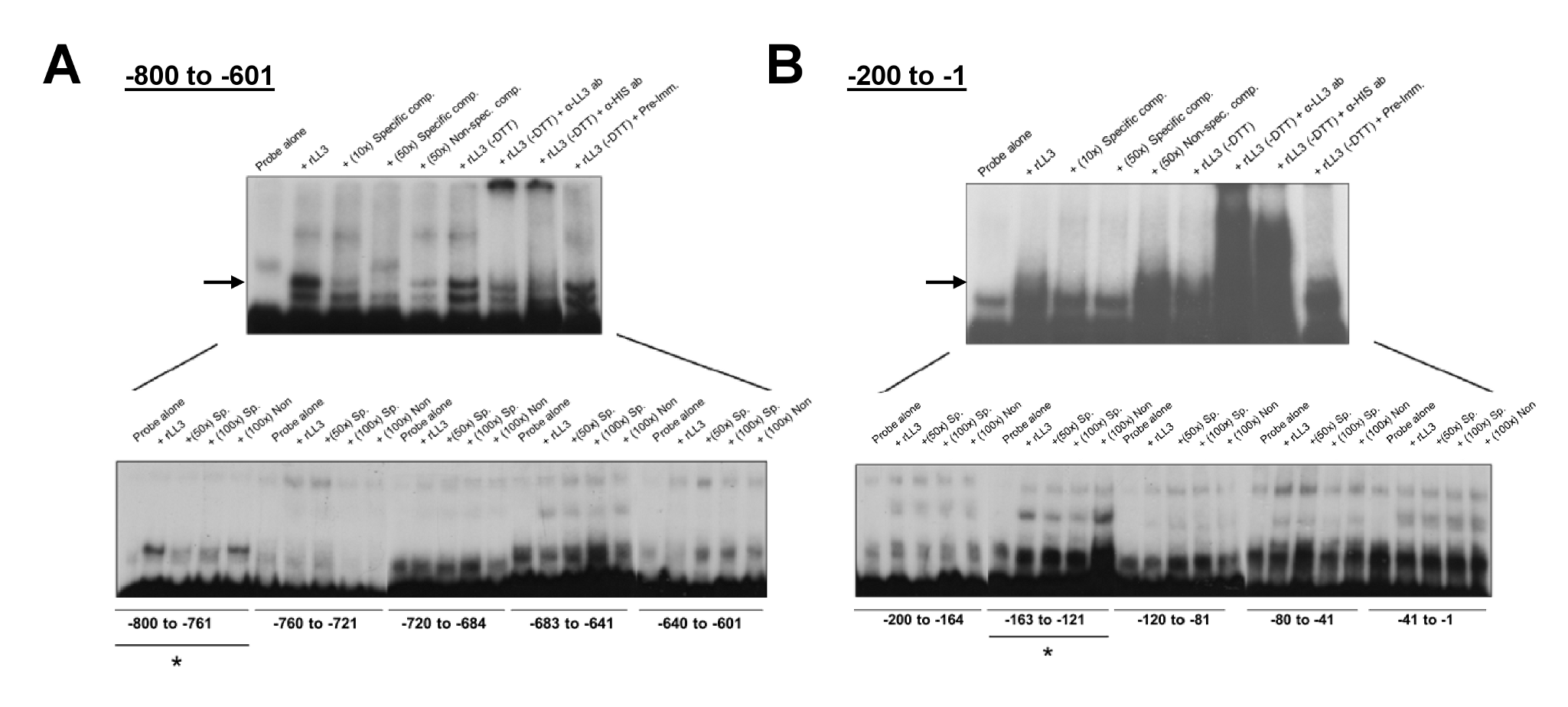

Supplement: Figure S5 — EMSA analysis of the SRPN6 promoter. The SRPN6 promoter was sequentially dissected by use of the Electrophoretic Mobility Shift Assay (EMSA) to identify regions capable of binding to the recombinant LL3 (rLL3) protein. One kilobase of the presumed SRPN6 promoter was divided into five 200-bp fragments that were PCR amplified, radiolabeled, and tested for their ability to bind to the rLL3 protein. The two 200-bp fragments that bound rLL3 were further analyzed under more stringent conditions. Specific binding to the regions −800 to −601 (A), and −200 to −1 (B) was demonstrated through the addition of specific and non-specific competitors and the presence of a super-shifted band in the presence of α-LL3 or α-His antibodies in the absence of DTT (-DTT)(upper panels). Double-stranded oligonucleotides of ∼40 bp each were used to identify the specific regions of rLL3 binding (lower panels). Analysis of the −800 to-601 region demonstrates strong, specific binding to the −800 to −761 fragment (A lower panel). Similar analysis demonstrates strong binding to the region from −163 to −121 within the −200 to −1 fragment (B lower panel). Components of each reaction are shown above each lane. Regions that display specific binding are underlined and denoted with an asterisk. Specific competitors are non-labeled fragments identical in sequence to the radioactive fragment in the assay, while non-specific competition was performed using a 200-bp rpS7 fragment or a 40-bp fragment from the Anopheles β2 tubulin gene. All primers used for PCR amplification of the 200-bp fragments or the syntheses of double-stranded oligonucleotides are shown in Table S1. (TIF) [file ppat.1002965.s005.tif]

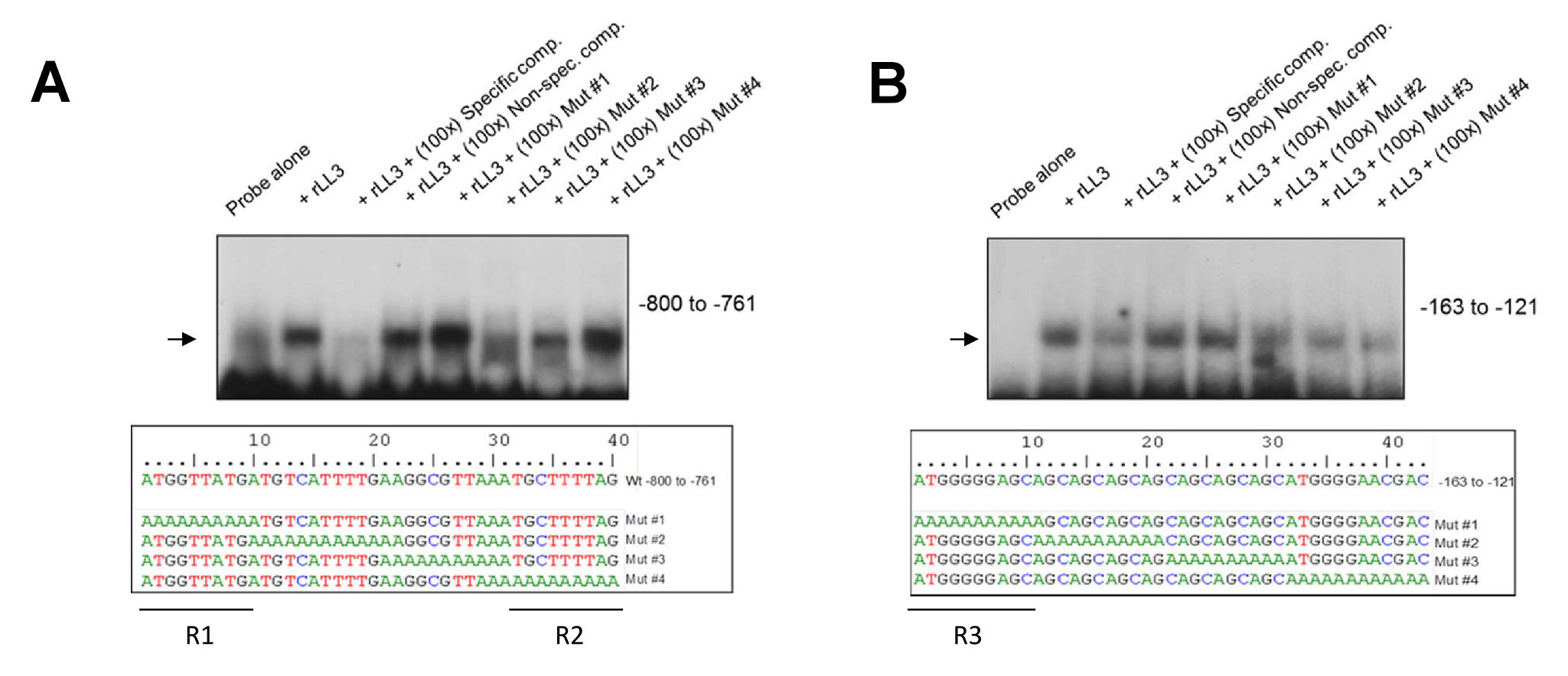

Supplement: Figure S6 — Mutational analysis to investigate LL3 target site specificity. To delineate the critical nucleotides involved in LL3-DNA interactions, the two ∼40 nucleotide regions −800 to −761 (A), and −163 to −121 (B) of the SRPN6 promoter that demonstrated specific rLL3 binding (Fig. S5) were mutated through the conversion of 10 bp stretches to adenosine residues. For each region, standard competition experiments were performed with specific, non-specific and mutated ds DNA fragments. To identify those DNA sequences involved in LL3-DNA interactions, mutated fragments that compete less efficiently than their wild type counterparts behave as non-specific competitors. In contrast, mutations not involved in LL3-DNA interactions have no effect, and compete as effectively as their wild type counterparts. The components of each reaction are shown above each lane, while the sequences of the wild type and mutated competitors are shown below. Regions of specific binding are denoted as regions R1-3. (TIF) [file ppat.1002965.s006.tif]
